# Gut microbiota and sirtuins in obesity-related inflammation and bowel dysfunction

**DOI:** 10.1186/1479-5876-9-202

**Published:** 2011-11-24

**Authors:** Shaheen E Lakhan, Annette Kirchgessner

**Affiliations:** 1 Global Neuroscience Initiative Foundation, Los Angeles, CA, USA; 2 School of Health and Medical Sciences, Seton Hall University, South Orange, NJ, USA

## Abstract

Obesity is a chronic disease characterized by persistent low-grade inflammation with alterations in gut motility. Motor abnormalities suggest that obesity has effects on the enteric nervous system (ENS), which controls virtually all gut functions. Recent studies have revealed that the gut microbiota can affect obesity and increase inflammatory tone by modulating mucosal barrier function. Furthermore, the observation that inflammatory conditions influence the excitability of enteric neurons may add to the gut dysfunction in obesity. In this article, we discuss recent advances in understanding the role of gut microbiota and inflammation in the pathogenesis of obesity and obesity-related gastrointestinal dysfunction. The potential contribution of sirtuins in protecting or regulating the circuitry of the ENS under inflamed states is also considered.

## Introduction

Obesity has dramatically increased during the past decades and has now reached epidemic proportions in both developed and developing countries. Even in Japan where the self-reported prevalence of obesity has remained consistently low over the last 30 years, obesity is now increasing in middle-aged adults and children [1,2] partly due to a western-style change in diet. The increase in obesity is associated with corresponding increases in type 2 diabetes, hypertension, cardiovascular disease and cancer [3]. Obesity is also associated with an increased incidence of gastrointestinal (GI) disorders [4] suggesting effects on the enteric nervous system (ENS), which controls virtually all gut functions (for review see [5]).

It is generally accepted that obesity is characterized by a low-grade chronic inflammation [6] in which pro-inflammatory cytokines play a pivotal role. The source of the inflammation is regarded as the adipose tissue itself. The adipose tissue of obese individuals, including adolescents, has been shown to produce higher levels of tumor necrosis factor (TNF)-α and interleukin (IL)-6 compared to lean individuals [7-10]. In animal models of diet-induced and genetic obesity increased production of IL-1, IL-6, TNF-α and Toll-like receptor (TLR) signaling in adipose tissue has also been reported [11-13]. In this review, the cause of the inflammation has been reevaluated and the GI tract as a potential source of inflammation and the role of gut microbiota are explored.

A high fat (HF) diet not only modulates the release of inflammatory mediators from adipocytes but also has a major impact upon the gut microbiota [14], the trillions of bacteria that normally reside within the human GI tract and upon fermentation of non-digestible carbohydrates generate short-chain fatty acids [15] and promote their absorption and storage as fat [16,17]. HF feeding in mice induced a low-grade inflammatory tone that was associated with changes in gut microbiota towards a decreased number of bifidobacteria [18-20], a group of bacteria, which has been shown to reduce lipopolysaccharide (LPS) levels in mice and to improve mucosal barrier function [21-23]. Interestingly, feeding obese mice with prebiotics and changing gut microbiota in favor of the *Bifidobacterium *spp. led to a significant improvement of gut permeability that correlated with lower portal plasma LPS levels and inflammatory tone (i.e., decreased circulating cytokines) [18,21]. The leakage of gut microbiota-derived LPS into the portal blood is a well-established mechanism of metabolic endotoxemia that triggers liver inflammation and oxidative stress [17,24]. The gut microbiota differs in composition between lean and obese individuals [14,25]. Moreover, alterations in gut microbiota seen in morbidly obese subjects are modulated by weight loss due to calorie restriction (CR) [26] or gastric bypass surgery and are correlated with a reduction in inflammatory state [27,28].

This article will discuss recent advances in understanding the role of gut microbiota and inflammation in the pathogenesis of obesity and obesity-related GI dysfunction. Since the silent information regulator (SIR) genes (sirtuins) are protective against obesity-induced inflammation and mediate, at least in part, the beneficial effects of CR (for review see [29,30]), the potential contribution of sirtuin signaling in the bowel under inflamed states is also considered.

### Gut microbiota

The human GI tract is dominated by anaerobic bacteria belonging to three bacterial phyla: Firmicutes, Bacteroidetes, and Actinobacteria [31]. Greater than 90% of the microbiota in a normal distal gut is represented by the Bacteroidetes and Firmicutes phyla [32]. The Firmicutes, which is the largest bacterial phylum, comprises over 200 genera of predominantly Gram-positive bacteria, including *Lactobacillus*, *Mycoplasma*, *Bacillus*, and *Clostridium *species. The Bacteroidetes phylum is composed of three large classes of Gram-negative bacteria: Cytophaga, Flavobacterium, and Bacteroidales. Members of the Bacteroidetes and Firmicutes phyla have been shown to be influenced by HF feeding and obesity [32-34]. The Actinobacterium phylum consists of Gram-positive bacteria and includes the genus *Bifidobacterium*, which is increased upon consumption of prebiotics, indigestible carbohydrates, which stimulate the growth of particular species of the microflora (for review see [35]).

Initial observations to suggest that the gut microbiota contribute to obesity were prompted by the observations of Gordon and colleagues [16,36]. Studies in leptin-deficient *ob/ob *mice showed a different proportion of the two dominating divisions, Bacteroidetes and Firmicutes compared to lean wild-type (WT) mice. Compared with lean littermates fed the same polysaccharide-rich diet, obesity was associated with a 50% reduction in Bacteriodetes and a proportional division-wide increase in Firmicutes in obese mice. To definitively demonstrate that gut microbiota composition is a cause and not a consequence of obesity, cecal microbiota from lean and obese mice were transplanted into the gut of germ-free (GF) mice. GF mice, raised from birth in sterile conditions, are significantly thinner than microbially colonized mice, despite eating the same amounts of food. Furthermore, after 2 weeks, conventionalization of GF mice with the cecal microbiota from normal mice produced a 57% increase in total body fat, a 2.3-fold increase in hepatic triglycerides, and much higher levels of leptin production and insulin resistance which was not dependent on an increase in chow consumption or changes in energy expenditure [37,38]. Evidence was obtained to suggest that an increased energy harvest from the diet contributed to obesity in host GF mice. This might be due to an increase in energy extraction due to bacterial fermentation of polysaccharides and also to the ability of the gut microbiota to upregulate fasting-induced adipose factor, a circulating lipoprotein lipase, which increases cellular uptake of fatty acids and triglyceride accumulation in adipocytes. Shotgun metagenomic analysis of the gut microbiome in obese and lean mice revealed an enrichment of genes involved in energy harvest in obese mice [34,38]. These included genes involved in sensing and degrading dietary polysaccharides, transporters of the resulting mono- and oligosaccharides, and genes involved in their intracellular metabolism. Thus, the microbiome of obese mice had increased fermentation capabilities resulting in increased levels of short chain fatty acids (SCFAs) in the cecum [38]. Similarly, the gut microbiome of obese individuals has increased fermentation capacity resulting in elevated SCFA production [38]. Interestingly, mice deficient in either of the SCFA receptors are leaner than their wild-type counterparts [39], further implicating SCFAs in the development of obesity. Currently, there is no consensus as to whether the gut microbiota plays a causative role in obesity or is modulated in response to the obese state itself or the diet in obesity. Further studies, especially on the regulatory role of SCFA in human energy homeostasis are needed to clarify the physiological consequences of an "obese style" microbiota and any putative dietary modulation of associated disease risk.

Data from human studies were generally consistent with the results from animal models. The first study describing qualitative changes of the gut microbiota in human obese subjects was published a few years ago [40]. In this study, the analysis of fecal samples of obese versus matched lean individuals showed a shift in bacterial phyla (lower Bacteroidetes and more Firmicutes). Interestingly the authors observed that after weight loss (following a fat restricted or a carbohydrate restricted low calorie diet) the ratio of Bacteroidetes to Firmicutes approached a lean type profile after 52 weeks [40]. In addition, humans who have undergone gastric bypass surgery for morbid obesity, have a microbial composition that is different from both obese and slim individuals [41]. A metagenomic study which included monozygotic and dizygotic twins concordant for leanness or obesity and their mothers, also showed that obesity was associated with a markedly reduced bacterial diversity, a relative depletion of Bacteroidetes, and a higher proportion of Actinobacteria [42].

The hypothesis of more specific modulation of the gut microbiota community in obesity (instead of those obtained at the wide *phylum *levels) is supported by several studies. Children with developmentally unusual gut microbiota appear to have predispositions to obesity [43] and another study found that the response of overweight adolescents to a diet and exercise weight-loss program was dependent on the initial gut microbiota prior to the treatment [26]. Moreover, differences in fecal microbiota were shown to predict overweight in children during the first year in life [43]. In a prospective study, *Bifidobacteria *spp. number was higher in children who exhibited a normal weight at 7 years than in children developing overweight. Moreover, they observed that the *Staphylococcus aureus *were lower in children who maintain a normal weight than in children becoming overweight several years later. The authors proposed that *S. aureus *may act as a trigger of low-grade inflammation, contributing to the development of obesity [43]. In agreement with these last findings, significant differences have been observed in gut microbiota composition according to the body weight gain during pregnancy [44]. They also found that the *Bifidobacterium *genus was present in higher numbers in normal-weight than in overweight women and also in women with lower weight gain during pregnancy [44]. The *Bifidobacterium *genus was also poorly represented in the fecal samples of diabetic patients compared with healthy individuals [45].

Similar results linking gut microbiota to obesity have been described in models of diet-induced obesity. In mice, ingestion of a HF diet resulted in an increase of Firmicutes and this was transmissible to lean GF recipient mice [34]. In addition, an increase in Bacteroidales and Clostridiales was found in rats fed a HF diet regardless of whether they exhibit either an obesity-prone or obesity-resistant phenotype. However, an increase in Enterobacteriales was seen in the microbiota of obesity-prone rats only [17]. Gut inflammation has been shown to promote the growth of Enterobacteriaceae [46]; therefore, an increase in this family may be a consequence of gut inflammation in the HF obesity prone mice rather than a cause. Taken together, these data demonstrate that it is the consumption of a HF diet rather than obesity that accounts for the change in the gut microbiota, but it is the development of inflammation that is associated with the appearance of hyperphagia and an obese phenotype.

Although humans are interested in manipulating microbiota to aid in weight loss, the food industry has been engaged for decades in manipulating microbiota to increase in weight gain through the use of low-dose antibiotics, usually called antibiotic growth promoters (AGPs) as feed additives [47]. Evidence from the food industry has shown that antibiotics, such as avoparcin (a vancomycin analogue) and Firmicute probiotics (e.g., Lactobacillus and Enterococcus) that modify the microbiota can act as growth promoters increasing the size and weight of farm animals [48]. Notably, a recent human clinical study showed significant weight gain can occur in humans after a six-week intravenous treatment of vancomycin plus gentamycin for infective endocarditis with a risk of obesity. Lactobacillus sp, a microorganism intrinsically resistant to vancomycin was found at higher concentration in the feces of obese patients [49]. In contrast, the absence of specific microbiota or its almost complete reduction with broad-spectrum antibiotics prevents or reverses HF-induced obesity [36,50]. Treatment with rifaximin (Xifaxan^®^, Salix Pharmaceuticals, Morrisville, NC, USA), a nonsystemic rifamycin-derived antibiotic that exhibits low gastrointestinal absorption while retaining potent antibacterial activity [51] for two weeks has recently been shown to provide significant relief of symptoms associated with IBS, such as bloating, abdominal pain, and loose or watery stools [52], which are also observed in obese individuals. Modulation of the gut microbiota with antibiotic therapy has been reported in obese mice. In addition, antibiotics reversed insulin resistance improving glycemic control [50].

### Gut inflammation and barrier function

It is now well established that obesity is an inflammatory condition and that "low grade chronic" inflammation, associated with insulin and leptin resistance exists in obese individuals [6]. The source of the inflammation is commonly regarded as the adipose tissue itself, which is known to produce inflammatory mediators [53]. However, the gut microbiota could also be a potential source of inflammation.

A HF diet is associated with the expression of two inflammatory biomarkers in the intestine, TNF-α and nuclear factor kappa B (NF-κB). The presence of gut bacteria is required for the induction of TNF-α and NF-κB since GF mice given a similar diet did not exhibit up-regulation of these pro-inflammatory markers [54]. Moreover, the observation that increases in intestinal TNF-α precede yet significantly correlate with body weight gain, body fat, and subsequent development of insulin resistance, supports a potential role of gut-derived TNF-α in the development of HF-induced obesity and obesity-related disease [54].

Recent work has shown that gut microbiota can initiate the inflammatory state of obesity through the activity of LPS, part of the outer membrane of Gram-negative bacteria that is released into the gut lumen when bacteria die. Cani et al. [18] reported that mice fed a HF diet present a chronic increase in circulating LPS, which they called "metabolic endotoxemia". The level of serum LPS is increased by about twice in obese, diabetic, or high-fat fed individuals, by processes involving an increase in chylomicron formation, a decrease in gut barrier integrity, and a decrease in alkaline phosphatase activity, which is the enzyme responsible for the cleavage of the LPS in the intestine [55,56,21].

LPS can trigger the inflammatory process by binding to the CD14 toll-like receptor-4 (TLR4) complex in the gut wall. When metabolic endotoxemia was reproduced by subcutaneous infusion of LPS, animals developed the same metabolic abnormalities induced by a HF diet, while LPS knock out (CD14*-/-*) mice were resistant to the effects of both HF diet and LPS infusion [57]. Moreover, chronic (4 week) administration of LPS in mice causes hyperphagia and an increase in adiposity and metabolic changes seen with ingestion of HF diet [21,57]. In a subsequent experiment, changes in gut microbiota composition induced by antibiotic treatment reduced the cecal content of LPS and improved measures of inflammation, such as macrophage infiltration of adipose tissue, closely correlating with an improvement in the obese phenotype in both HF fed and *ob/ob *mice [19].

Activation of TLR4 causes the secretion of IL-6 and TNF-α, supporting the role of LPS in triggering the downstream inflammatory processes associated with obesity, such as metabolic disease [20,57]. Ingestion of a HF diet induced a significant postprandial elevation of LPS, accompanied by an increased mononuclear cell expression of TLR-4, NF-ΚB and suppressor of cytokine signaling-3 (SOCS-3), an adipokine involved in insulin resistance [58].

Interestingly, mice genetically deficient in TLR-5 have an altered gut microbiota composition that correlates with obesity and several features of the metabolic syndrome including hyperlipidemia, hypertension, and insulin resistance, which could at least in part be attributed to increased food consumption [59]. In contrast to the *ob*/*ob *mouse model of obesity, which is characterized by a phylum-level shift in Bacteroidetes and Firmicutes, the TLR5-deficient mice exhibit altered abundance in over one-hundred specific bacterial phylotypes. A direct causal relationship between the altered gut microbiota and obesity was demonstrated by gut microbiota transplants where GF mice receiving the gut microbiota from TLR-5-deficient mice gained significantly more weight compared to mice that received gut microbiota from wild-type mice [59]. These results support the emerging view that the gut microbiota contributes to obesity and suggest that malfunction of the innate immune system may promote the development of obesity-related disorders such as metabolic syndrome.

Mucosal barrier function is maintained by several interrelated systems, including mucous secretion, chloride and water secretion, and binding together of epithelial cells at their apical junctions by tight junction (TJ) proteins. The disruption of the TJ complex leads to leakage of water and proteins into the lumen, as described in relapsing diarrhea, and to the translocation of intraluminal solutes, such as bacterial endotoxins (LPS), into the system circulation [60]. Activation of TLR4 has previously been shown to alter the TJ complex and increase intestinal permeability [61]. Modulation of gut bacteria following a HF diet strongly increases intestinal permeability, by reducing the expression of genes coding for two intestinal TJ proteins, ZO-1 and occluding [19]. Alteration in occludin distribution has also been reported *in vitro *on epithelial cells stimulated with pro-inflammatory cytokines; thus, occludin was chosen as a marker of TJ disruption [24,62].

Since aberrant gut microbiota and a "leaky" mucosal barrier are found in obesity they offer potential targets for intervention that would include modulation of the intestinal microbiota to correct an imbalance, as well as tightening of interepithelial junctions. Enhancement of barrier function by probiotic bacteria has been observed both in *in vitro *models and *in vivo *in the whole animal [63]. Probiotics are live microorganisms that have a beneficial effect on the intestinal mucosa via several proposed mechanisms that include inhibition of the mucosal adhesion of pathogens, improvement of the barrier function of the epithelium, and alteration of the immune activity of the host. They may also regulate intraluminal fermentation and stabilize the intestinal microbiota [64]. Probiotic bacteria are *Lactobacilli spp*., certain types of *Streptococcus*, and *Bifidobacteria spp*., but also other non-pathogenic bacilli such as *E. coli*-Nisle 1917 and yeasts such as *Saccharamyces boulardii*. They secrete short chain fatty acids, an action that results in decreased luminal pH and production of bactericidal proteins. Butyric acid, a byproduct of bacterial fermentation of fiber, has been shown to nourish colonic enterocytes, enhancing mucosal integrity [65].

Researchers have demonstrated the utility of probiotics for obesity in HF fed mice [57,66], which is associated with a decrease in the number of *Bifidobacteria *[21]. An increase in *Bifidobacteria *in *ob/ob *mice was associated with a significant improvement of gut permeability measured *in vivo*; this improvement was linked to an increase in TJ mRNA expression and protein distribution [21]. In addition, the rise in *Bifidobacteria *was correlated with a decrease in plasma LPS concentrations and therefore, a significant reduction in markers of oxidative and inflammatory stress [21]. Potential beneficial effects of probiotics on gut motility via a direct action on the ENS or epithelial cells have also been demonstrated [67]. In experimental studies, *Lactobacillus *inhibited post-infective intestinal hypercontractility through an unidenfied, heat-labile fermentation-product and by blocking calcium-dependent potassium channels [68,68,69]. Supernatant of *Escherichia coli *Nissle 1917 enhanced human colonic motility *in vitro *and acute exposure of colonic mucosa to *Lactobacillus rhamnosus *GG significantly reduced the acetylcholine-stimulated human colonic contractions in a dose- and time-dependent manners [70]. Administration of *L. reuteri *altered the motility of *ex vivo *colonic segment of rat; it decreased the amplitudes of contractions and increased intraluminal fluid filling pressure thresholds for evoking pressure pulses [71]. Overall, probiotics will likely have an emerging therapeutic role in preventing and treating obesity and obesity-related inflammation.

### Gut dysfunction and obesity

Many obese individuals report symptoms suggestive of gut dysfunction including bloating, abdominal pain and diarrhea [4,72,73]. Bloating and upper abdominal pain increased in frequency with increasing body mass index (BMI). There was also a significant positive relationship between BMI and diarrhea. In contrast, no significant relationship was observed between BMI and constipation, even though it was more frequent in obese patients [4]. Potential mechanisms to explain the increased bowel frequency would be rapid gastric emptying, which has been reported in some groups of obese patients [74], and increased colonic motility, although the latter has not yet been demonstrated.

Obesity is associated with gastroesophageal reflux disease (GERD), nonalcoholic fatty liver disease (NAFLD), and increased occurrence of cholelithiasis [75,76]. GERD has been shown to be more common in obese patients than in those with a BMI within normal range, and an increase in the BMI above the 95 percentile for age and gender is a significant risk factor for GERD [77,78]. Also, a higher BMI is associated with more frequent and more severe heartburn and regurgitation in patients with GERD and increasing BMI is a strong predictor of heartburn during sleep [79,80].

In patients with irritable bowel syndrome (IBS), heartburn was more likely to be present in subjects with obesity, and epigastric pain and nausea, were also more common in overweight patients with IBS. However, in an adjusted log linear model, no significant interaction was found between BMI and any other studied symptom and heartburn was found to be independent of IBS [81].

An important and well described correlation also exists between obesity and colorectal cancer [82]. Epidemiologic data have shown that obesity independently increases colorectal cancer risk, particularly in males, but the mechanisms are poorly understood [83]. Serum leptin level in colon cancer patients who were overweight or obese were significantly higher compared to patients with normal weight [84]. mRNA levels of the novel inflammatory factors lipocalin-2, chitinase-3 like-1 and osteopontin are increased in human visceral adipose tissue of individuals with colon cancer [85]. Leptin upregulates pro-inflammatory cytokines in discrete cells within the mouse colon [86]. IL6, IL1β and CXCL1 were upregulated by leptin and localized to discrete cells in gut epithelium, lamina propria, muscularis and at the peritoneal serosal surface.

Diet-induced weight loss in obese individuals reduces colorectal inflammation and greatly modulates inflammatory and cancer-related gene pathways [87]. After weight-loss, rectosigmoid biopsies showed a 25-57% reduction in TNF-α, IL-1β, and MCP-1 concentrations. Gene arrays showed dramatic down-regulation of pro-inflammatory cytokine and chemokine pathways, prostaglandin metabolism, oxidative stress pathways and the transcription factor CREB. These data imply that obesity is accompanied by inflammation in the colorectal mucosa and that weight loss reduces this inflammatory state and may thereby lower colorectal cancer risk [87].

Obesity predicts persistence of abdominal pain in children with functional gastrointestinal disorders [88]. Obese children (mean age 13 years) were more likely to have abdominal pain, higher intensity of pain, higher frequency of pain, school absenteeism and disruption of daily activities than non-obese children [88]. Obesity is more common in children with celiac disease, a T cell-mediated chronic autoimmune enteropathy occurring in genetically susceptible individuals, and manifested by a permanent intolerance of gluten-containing products [89]. The most common presenting symptoms among obese patients were abdominal pain, diabetes, and diarrhea. Symptoms improved in all patients on a gluten-free diet.

### Role of the ENS

Under both physiological and pathological conditions, the ENS, the intrinsic innervation of the bowel, regulates intestinal mucosal function and coordinates the activity of the GI tract. The ENS is a component of the autonomic nervous system with the unique ability to function independently from the CNS (for review, see [90]). Enteric ganglia are organized into two major ganglionated plexuses, namely the myenteric (Auerbach's) and submucosal (Meissner's) plexus, and contain a variety of functionally distinct neurons, including primary afferent neurons, interneurons, and motor neurons, synaptically linked to each other in microcircuits. While the myenteric plexus mainly regulates intestinal motility, the submucosal plexus together with nerve fibers in the lamina propria are involved in regulating epithelial transport. These nerves form networks within the lamina propria of both crypts and villi with the terminal axons in close contact with the basal lamina, an ideal position not only to affect epithelial cell functions but also to detect absorbed nutrients and antigens. Substances released from epithelial cells may act on nerve terminals to change the properties of enteric neurons and cause peripheral sensitization. Consequently, permanent or even transient structural alterations in the ENS disrupt normal GI function.

The ENS is increasingly recognized as a regulatory housekeeper of the epithelial barrier integrity, in addition to its ascribed immunomodulatory potential (for review see [5]). Inflammation affects both epithelial integrity and barrier function and, in turn, loss of barrier function perpetuates the inflammatory condition. Several studies have demonstrated structural changes within enteric ganglia in gut inflammation (see [91,92] for review). For example, damage to axons has been observed in the inflamed human intestine in episodes of inflammatory bowel disease (IBD) [93]. Other changes that occur in the ENS during inflammation include altered neurotransmitter synthesis, content, and release, changes in glial and myenteric cell numbers and a myenteric ganglionitis associated with infiltrates of lymphocytes, plasma cells and mast cells [94-96]. In fact, experimental data show that gut inflammation, even if mild, could lead to persistent changes in GI nerve and smooth muscle function, resulting in dysmotility, hypersensitivity, and dysfunction (for review see [91,92]). Furthermore, alterations in gut function were observed even after the resolution of an acute intestinal inflammation [97-99]. Thus, the breakdown of mucosal barrier function as occurs in obesity could cause alterations in the patterns of gut motility, abnormal secretion, and changes in visceral sensation that contributes to symptom generation. In a rodent model of diet-induced obesity the secretomotor function of submucosal neurons was compromised, which may lead to an altered host defense with a resultant change in intestinal flora contributing to the maintenance of obesity [100]. The breakdown of mucosal barrier function may at least partially explain the link between obesity and gut dysfunction. Whether the persistent alterations in GI motility observed in many obese patients are due to inflammation-related changes in the properties of enteric nerves is not known. However, probiotic lactic acid producing-bacteria have been shown to prevent and alleviate GI disturbances and to normalize the cytokine profile which might be of an advantage for patients suffering from obesity [101].

### Role of sirtuins

In the past decade, a novel class of regulators, the silent regulator 2 (SIR2), has been linked to metabolic regulation and aging and shown to mediate CR-induced longevity in yeast and possibly other organisms (for review see [29]). Mammalian sirtuins are conserved with seven genes (SIRT1-7) homologous to the yeast *Sir2 *gene. Like their yeast homologs, the mammalian sirtuins are class III histone deacetylases and require NAD(+) as a cofactor to deacetylate substrates ranging from histones to transcriptional regulators. The nuclear sirtuins (SIRT1, SIRT6, and SIRT7), the mitochondrial sirtuins (SIRT3, SIRT4, and SIRT5), and the cytosolic sirtuin (SIRT2) regulate diverse metabolic functions. For example, SIRT6 functions in genomic stability and transcriptional control of glucose metabolism and its deficiency (SIRT6-/-) causes a lethal hypoglycemia [102,103]. SIRT6 is highly expressed in the CNS and mice overexpressing SIRT6 are protected against diet-induced obesity [104]. In contrast, neural-specific SIRT6 knockout mice become obese during adult life [105], further highlighting the importance of SIRT6 in the context of nutrient metabolism. SIRT3 is an integral regulator of mitochondrial function and its depletion results in hyperacetylation of critical mitochondrial proteins that protect against hepatic lipotoxicity under conditions of nutrient excess. Livers of mice fed on a HF diet had reduced SIRT3 activity [106].

The beneficial effect of CR on aging and various metabolic disorders is dependent on the activation of SIRT1 and can be mimicked by resveratrol, a product present in grape skin and red wine, which activates the SIRT1 enzyme [107,108]. Mild to moderate red wine consumption has anti-inflammatory properties, and can reduce the risk of cardiovascular disease and cancer. The resveratrol content in red wine is often cited to account for this "French paradox." Evidence for a role of sirtuins in obesity comes from emerging understanding of the regulatory role sirtuins play in metabolic pathways and adaptations linked with obesity and aspects of metabolic syndrome. These include the expression of adipocyte cytokines (adipokines), the maturation of fat cells, insulin secretion, modulation of plasma glucose levels, cholesterol and lipid homeostasis and mitochondrial energy capacity [29].

SIRT1, for example, is involved in regulating the expression of adipokines such as adiponectin and TNF-α, has been linked to hypothalamic control of energy balance, plays a role in adipogenesis, and is involved in the regulation of lipolysis and fatty acid mobilization in response to fasting.[29] Evidence from animal studies in which sirtuins are under- or over-expressed, and from limited human evidence, also suggest a role for sirtuins in obesity. Existing evidence on resveratrol suggests that this compound may have sirtuin-mediated anti-obesity effects [109].

Fasting leads to the up-regulation of SIRT1 in adipose tissue of mice, pigs and humans whereas decreased SIRT1 expression is associated with obesity [29]. In both *db/db *and obese HF fed mice SIRT1 expression is low in adipose tissue [110]. Circumstances that result in SIRT1 under-expression in adipose tissue enhanced adipogenesis, while circumstances that promote fat SIRT1 over-expression were characterized by attenuated adipogenesis and increased lipolysis [111]. Consistent with this idea, lean women had more than twofold higher SIRT1 expression compared to obese women [112]. Benefits of SIRT1 over-expression also included less inflammation, better glucose tolerance, and almost complete protection against hepatic steatosis, suggesting that SIRT1 plays an important role in obesity-associated metabolic adverse effects. Consequently, if activation of SIRT1 results in loss of body fat without decreasing caloric intake, this could open the door for novel treatment and prevention strategies for obesity and related diseases.

Genetic variation in SIRT1 is related to BMI and risk of obesity in humans [113,114]. In a recent Belgian case/control study of 1,068 obese patients (BMI ≥ 30 kg/m^2^) and 313 normal weight control subjects, a SIRT1 single nucleotide polymorphism (SNP) associated with visceral obesity parameters in obese men but not women [113]. In two large and independent Dutch Caucasian populations, two common variants in *SIRT1 *were associated with lower BMI. Carriers of these two common genetic variants had 9-11% decreased risk of being overweight and 13-18% decreased risk of being obese compared with noncarriers [114].

SIRT1 has recently been implicated in the regulation of obesity-related inflammation. Zhu et al. [115] demonstrated that resveratrol, a SIRT1 activator, decreased TNF-α-induced MCP-1 secretion in 3T3-L1 adipocytes. Pfluger et al. [116] showed over-expression of SIRT1 in mice resulted in a lower level of IL-6 and TNF-α in the serum of transgenic mice fed a HF diet and an attenuated response to TNF-α-induced NF-κB activation in transgenic mouse embryonic fibroblasts. Resveratrol regulates human adipocyte number and down-regulates the expression and secretion of IL-6 and IL-8 from mammalian adipocytes in a SIRT1-dependent manner [117]. Furthermore, mice fed a diet supplemented with 0.4% resveratrol for 10 weeks showed significantly lower body weight and visceral fat-pad weights than HF diet fed mice. Resveratrol significantly attenuated the HF diet-induced up-regulation of a number of pro-inflammatory cytokines such as TNF-α and IL-6, and their upstream molecules, including TLR4 and NF-κB in epididymal adipose tissues of mice [12]. Thus, increased SIRT1 activity appears to be anti-inflammatory in mice and resveratrol may improve obesity-induced inflammation and add to the potential of this dietary polyphenol in the control of obesity. In contrast, inhibition of SIRT1 appears to be pro-inflammatory. Studies using small interfering RNA (siRNA) to knock down SIRT1 reported an increase in TNF-α-induced MCP-1 and other pro-inflammatory genes in 3T3-L1 adipocytes [118]. Taken together, these data demonstrate that a decrease in SIRT1 activity increases activation of NF-κB and transcription of pro-inflammatory mediators. These results have important clinical implications and may thus provide a valuable new strategy for treatment of obesity and its related diseases.

In addition to adipose tissue, SIRT1 is highly expressed in the hypothalamus where it appears to be involved in regulating energy homeostasis, food intake and body weight [108,119]. Fasting up-regulates hypothalamic SIRT1 expression, which is associated with a fasting-induced increase in hunger, and presumably part of the complex adaptations against CR-induced weight loss [119]. Conversely, pharmacological inhibition of hypothalamic SIRT1 decreases food intake and body weight gain in rodents [120], suggesting that hypothalamic SIRT1 decreases food intake and body weight gain in rodents. Lack of SIRT1 in pro-opiomelanocortin (POMC) neurons causes hypersensitivity to HF obesity [121].

SIRT1 is also expressed in the gut where it exerts anti-inflammatory effects in acute intestinal inflammation and suppresses intestinal tumorigenesis and colon cancer associated with colitis [122-124]. Rats fed with 1 mg of resveratrol/kg/day (a human equivalent dose) for 25 days, and in the last five days, 5% DSS to induce colitis, displayed increased lactobacilli and bifidobacteria as well as a reduced increase in enterobacteria upon DSS treatment. In addition, resveratrol significantly protected the colonic mucosa architecture, reduced body weight loss, diminished the induced anemia and reduced systemic inflammatory markers possibly via the down-regulation of NF-κB [124]. Also, resveratrol up-regulated SIRT1 expression in the mucosa and mitigated the increase in the number of mucosal CD4+ T cells suggesting that resveratrol may exert its anti-inflammatory effects by modulating activated immune cells [123]. Thus, activation of SIRT1 maintains gut barrier function, which is compromised in obesity [125] and through its regulation of gut inflammation controls colitis and colon cancer, which are also more prevalent in obese individuals [126].

**Figure 1:**
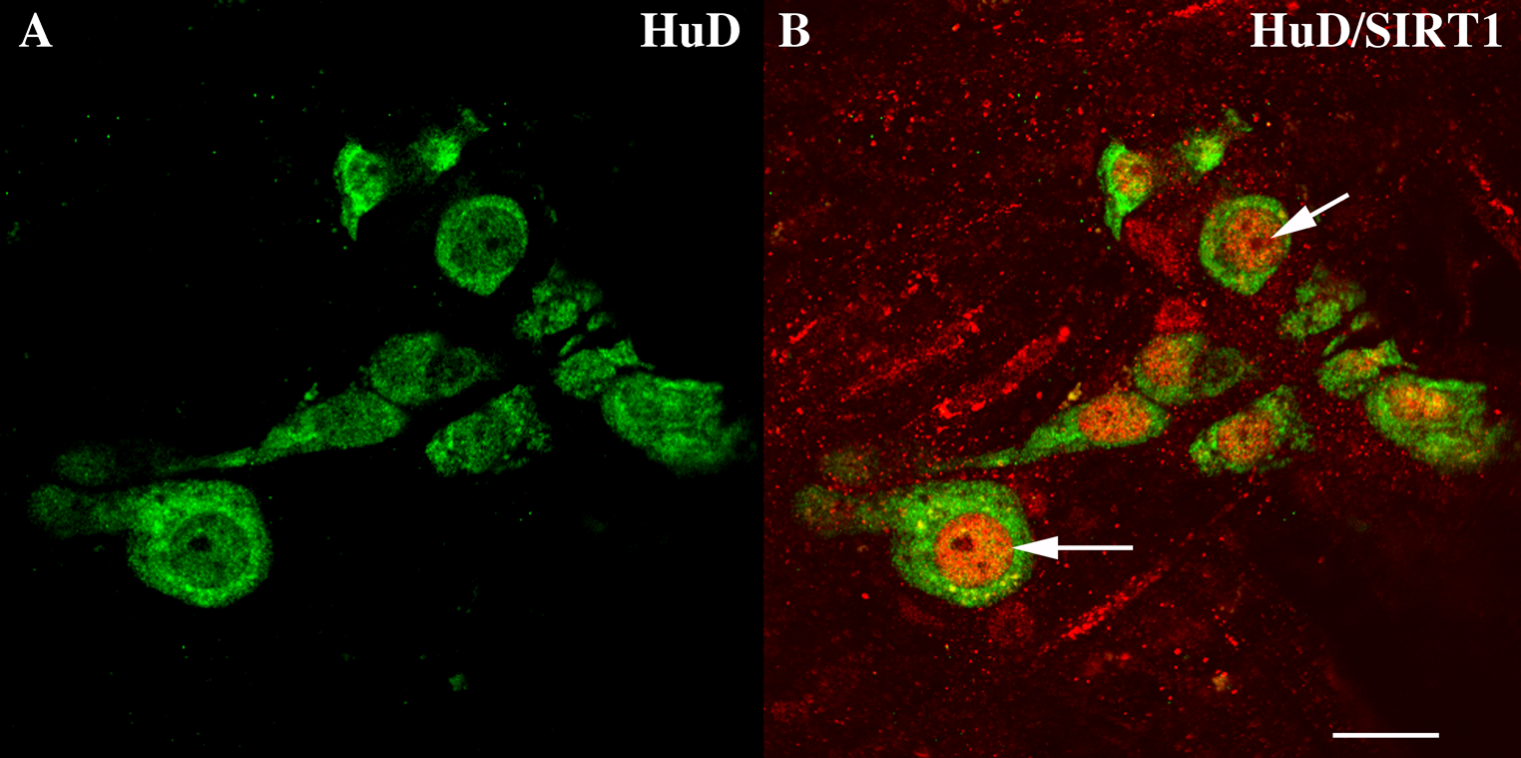
**Immunohistochemical localization of the class III histone deacetylase SIRT1 (Sir2) in the murine enteric nervous system**. **A**. Confocal image of a whole mount preparation of colon stained with a goat antibody to the neuronal marker human neuronal protein (HuD; 1:100; Santa Cruz; sc-5977; green). HuD immunoreactivity is displayed by neurons in a myenteric ganglion. **B**. Double label confocal image of the same area depicted in **A **stained with an antibody to HuD and a SIRT1-specific antibody made in rabbit (1:500; Abcam Inc. Cambridge MA; ab 16640). Myenteric neurons display both HuD (green) and nuclear SIRT1 immunoreactivity (red). For whole-mount preparations, segments of colon were cut along the mesenteric border and the resulting sheet of gut was pinned flat, mucosal side up, in a silicone elastomer (Sylgard, Dow Corning, Midland, MI)-coated dish. The tissue was fixed for 3 hours with 4% paraformaldehyde in 0.1 M phosphate buffer (pH 7.4). After fixation, the preparations were washed in phosphate-buffered saline (PBS) for 1 hour and whole-mount preparations of longitudinal muscle with adherent myenteric plexus (LMMP) were generated as previously described [128]. Non-specific binding was blocked by incubating the preparations with 6% (v/v) normal horse serum, with Triton X-100 (0.5%), in PBS for 60 minutes. The preparations were then exposed for 24 h to primary antibodies at 4°C. After washing with PBS, sites of bound primary antibodies were detected by incubation with donkey anti-rabbit or donkey anti-goat secondary antibodies coupled to DyLight™ 549 (1:400; Jackson ImmunoResearch Labs.West Grove, PA) or DyLight™ 488 (1:400; Jackson ImmunoResearch Labs.) for 3 hours. Confocal images were obtained using an Olympus FluoView FV300 confocal microscope. Scale bar, 30 μm.

The ENS also contains sirtuins. We have shown for the first time that neurons in the murine colon display SIRT1 immunoreactivity (Figure 1). The cellular localization of SIRT1 is predominantly nuclear and displayed by neurons in both the submucosal and myenteric plexus consistent with areas strongly compromised by aging [127]. These findings propose a role for SIRT1 in gut motility and secretion and suggest a previously unrecognized role of enteric SIRT1 in regulating energy homeostasis. Moreover, activation of enteric sirtuin pathways could offer a therapeutic approach to treat obesity-related gut dysfunction. Clearly, further research is required to explore the role of sirtuin proteins in enteric neurobiology during normal and inflamed states.

## Conclusions

Obesity is considered a major public health concern globally as it predisposes to a number of chronic human diseases. Recent studies report an aberrant gut microbiota in obese individuals and that gut microbial metabolic activities, especially fermentation can impact on a number of mammalian physiological functions linked to obesity. Those data suggest that specific changes in the gut microbiota characterize the obese state and associated metabolic diseases, including diabetes. Gut microbiota, which affect barrier function also modulate the activity of the ENS, a key player in gut dysfunction. Since aberrant gut microbiota and a "leaky" mucosal barrier are found in obesity they offer potential targets for intervention that would include modulation of the intestinal microbiota to correct an imbalance, as well as tightening of interepithelial junctions. However, we may not conclude, from the papers published until now, that targeting one specific bacterial target is sufficient to get an improvement of a complex disease such as obesity. Future studies using newly developed techniques to evaluate the gut microbiota in obese patients and/or animal models of obesity are certainly needed. In addition, future research should focus on the role of sirtuin proteins in the gut and their function in obesity and gut dysfunction. Studies suggest that resveratrol acts as an anti-inflammatory agent in the gut by targeting the SIRT1 gene. Thus, resveratrol may be a therapeutic target against obesity-related inflammation and gut dysfunction.

## List of abbreviations

Body mass index: BMI; calorie restriction: CR; enteric nervous system: ENS; gastroesophageal reflux disease: GERD; gastrointestinal: GI; germ-free: GF; high-fat diet: HF; inflammatory bowel disease: IBD; irritable bowel syndrome: IBS; interleukin: IL; interferon: IFN; lipopolysaccharide: LPS; nonalcoholic fatty liver disease: NAFLD; nuclear factor kappa B: NF-κB; pro-opiomelanocortin: POMC; short chain fatty acid: SCFA; silent information regulator: SIR; silent information regulator genes: sirtuins; small interfering RNA: siRNA; suppressor of cytokine signaling-3: SOCS-3; tight junction: TJ; Toll-like receptor: TLR; tumor necrosis factor: TNF; wild type: WT.

## Competing interests

The authors declare that they have no competing interests.

## Authors' contributions

All authors participated in the preparation of the manuscript, and read and approved the final manuscript.

## References

[B1] Matsushita Y Yoshiike F Yoshita K Takimoto H Trends in childhood obesity in Japan over the last 25 years from the national nutrition survey Obesity Res 2004 12 205 214 10.1038/oby.2004.27 14981212

[B2] Sasai H Sairenchi T Hiroyasu I Irie F Otaka E Tanaka K Ota H Muto T Relationship between obesity and incident diabetes in middle-aged and older Japanese adults: The Ibaraki prefectural health study Mayo Clin Proc 2010 85 36 40 10.4065/mcp.2009.0230 PMC2800296 20042559

[B3] Field AE Coakley EH Must A Spadano JL Laird N Dietz WH Rimm E Colditz GA Impact of overweight on the risk of developing common chronic diseases during a 10-year period Arch Intern Med 2001 161 1581 1586 10.1001/archinte.161.13.1581 11434789

[B4] Delgado-Aros S Locke GR Camilleri M Talley NJ Fett S Zinsmeister AR Melton LJ Obesity is associated with increased risk of gastrointestinal symptoms: a population-based study Am J Gastroenterol 2004 99 1801 1806 10.1111/j.1572-0241.2004.30887.x 15330922

[B5] Snoek SA Verstege MI Boeckxstaens GE van den Wijngaard RM de Jonge WJ The enteric nervous system as a regulator of intestinal epithelial barrier function in health and disease Expert Rev Gastroenterol Hepatol 2010 4 637 651 10.1586/egh.10.51 20932148

[B6] Hotamisligil GS Inflammation and metabolic disorders Nature 2006 444 860 867 10.1038/nature05485 17167474

[B7] Kern PA Ranganathan S Li C Wood L Ranganathan G Adipose tissue tumor necrosis factor and interleukin-6 expression in human obesity and insulin resistance Am J Physiol Endocrinol Metab 2001 280 E745 E751 10.1152/ajpendo.2001.280.5.E745 11287357

[B8] Syrenicz A Garanty-Bogachka B Syrenicz M Gebala A Walczak M Low-grade systemic inflammation and the risk of type 2 diabetes in obese children and adolescents Neuro Endocrinol Lett 2006 27 453 458 16892000

[B9] Panagiotakos DB Pitsavos C Yannakoulia M Chrysohoou C Stefanadis C The implication of obesity and central fat on markers of chronic inflammation: The ATTICA study Atherosclerosis 2005 183 308 315 10.1016/j.atherosclerosis.2005.03.010 16285994

[B10] Viser M Bouter LM McQuillan GM Wener MH Harris TB Low-grade systemic inflammation in overweight children Pediatrics 2001 107 E13 10.1542/peds.107.1.e13 11134477

[B11] Brake DK Smith EO Mersmann H Smith CW Robker RL ICAM-1 expression in adipose tissue: effects of diet-induced obesity in mice Am J Physiol Cell Physiol 2006 291 C1232 1239 10.1152/ajpcell.00008.2006 16807303

[B12] Kim SJ Choi Y Choi YH Park T Obesity activates toll-like receptor-mediated proinflammatory signaling cascades in the adipose tissue of mice J Nutr Biochem 2011 10.1016/j.jnutbio.2010.10.012 21414767

[B13] Xu H Barnes GT Yang Q Tan G Yang D Chou CJ Sole J Nichols A Ross JS Tartaglia LA Chronic inflammation in fat plays a crucial role in the development of obesity-related insulin resistance J Clin Invest 2003 1123 1821 1830 10.1172/JCI19451 PMC296998 14679177

[B14] DiBaise JK Zhang H Crowell MD Krajmalnik-Brown R Decker GA Rittmann BE Gut microbiota and its possible relationship with obesity Mayo Clin Proc 2008 83 460 469 10.4065/83.4.460 18380992

[B15] Conterno L FF Viola R Tuohy KM Obesity and the gut microbiota: does up-regulating colonic fermentation protect against obesity and metabolic disease? Genes Nutr 2011 6 241 260 10.1007/s12263-011-0230-1 PMC3145060 21559992

[B16] Bäckhed F Ding H Wang T Hooper LV Koh GY Nagy A Semenkovich CF Gordon JI The gut microbiota as an environmental factor that regulates fat storage Proc Natl Acad Sci 2004 101 15718 15723 10.1073/pnas.0407076101 PMC524219 15505215

[B17] de La Serre CB Ellis CL Lee J Hartman AL Rutledge JC Raybould HE Propensity to high-fat diet-induced obesity in rats is associated with changes in the gut microbiota and gut inflammation Am J Physiol Gastrointest Liver Physiol 2010 299 G440 448 10.1152/ajpgi.00098.2010 PMC2928532 20508158

[B18] Cani PD Neyrinck AM Fava F Knauf C Burcelin RG Tuohy KM Gibson GR Delzenne NM Selective increases of bifidobacteria in gut microflora improve high-fat-diet-induced diabetes in mice through a mechanism associated with endotoxaemia Diabetologia 2007 50 2374 2383 10.1007/s00125-007-0791-0 17823788

[B19] Cani PD Bibiloni R Knauf C Waget A Neyrinck AM Delzenne NM Burcelin R Changes in gut microbiota could control endotoxemia-induced inflammation in high-fat diet-induced obesity and diabetes in mice Diabetes 2008 57 1470 1481 10.2337/db07-1403 18305141

[B20] Cani PD Delzenne NM Amar J Burcelin R Role of gut microflora in the development of obesity and insulin resistance following high-fat diet feeding Pathol Biol (Paris) 2008 56 305 309 10.1016/j.patbio.2007.09.008 18178333

[B21] Cani PD Possemiers S Van de Wiele T Guiot Y Everard A Rottier O Geurts L Naslain D Neyrinck A Lambert DM Changes in gut microbiota control inflammation in obese mice through a mechanism involving GLP-2-driven improvement of gut permeability Gut 2009 58 1091 1103 10.1136/gut.2008.165886 PMC2702831 19240062

[B22] Griffiths EA Duffy LC Schanbacher FL Qiao H Dryja D Leavens A Rossman J Rich G Dirienzo D Ogra PL In vivo effects of bifidobacteria and lactoferrin on gut endotoxin concentration and mucosal immunity in Balb/c mice Dig Dis Sci 2004 49 579 589 10.1023/b:ddas.0000026302.92898.ae 15185861

[B23] Wang Z Xiao G Yao Y Guo S Lu K Sheng Z The role of bifidobacteria in gut barrier function after thermal injury in rats J Trauma 2006 61 650 657 10.1097/01.ta.0000196574.70614.27 16967002

[B24] Brun P Castagliuolo I Di Leo V Buda A Pinzani M Palu G Martines D Increased intestinal permeability in obese mice: new evidence in the pathogenesis of nonalcoholic steatohepatitis Am J Physiol Gastrointest Liver Physiol 2007 292 G518 G525 10.1152/ajpgi.00024.2006 17023554

[B25] Scarpellini E Campanale M Leone D Purchiaroni F Vitale G Lauritano EC Gasbarrini A Gut microbiota and obesity Intern Emerg Med 2010 5 Suppl 1 S53 56 10.1007/s11739-010-0450-1 20865475

[B26] Santacruz A Marcos A Wärnberg J Martí A Martin-Matillas M Campoy C Moreno LA Veiga O Redondo-Figuero C Garagorri JM Interplay between weight loss and gut microbiota composition in overweight adolescents Obesity 2009 17 1906 1915 10.1038/oby.2009.112 19390523

[B27] Chaudhri O Wynne K Bloom SR Can gut hormones control appetite and prevent obesity? Daibetes Care 2008 31 Suppl 2 S284 S289 10.2337/dc08-s269 18227498

[B28] Furet JP Kong LC Tap J Poitou C Basdevant A Bouillot JL Mariat D Corthier G Doré J Henegar C Differential adaptation of human gut microbiota to bariatric surgery-induced weight loss: links with metabolic and low-grade inflammation Diabetes 2010 59 3049 3057 10.2337/db10-0253 PMC2992765 20876719

[B29] Kelly G A review of the sirtuin system, its clinical implications, and the potential role of dietary activators like resveratrol: part 2 Alt Med Rev 2010 15 313 338 21194247

[B30] Schug TT Li X Sirtuin 1 in lipid metabolism and obesity Ann Med 2011 10.3109/07853890.2010.547211 PMC3173813 21345154

[B31] Greiner T Backhed F Effects of the gut microbiota on obesity and glucose homeostasis Trends Endocrinol Metab 2011 10.1016/j.tem.2011.01.002 21353592

[B32] Hildebrandt MA Hoffmann C Sherrill-Mix SA Keilbaugh SA Hamady M Chen YY Knight R Ahima RS Bushman F Wu GD High-fat diet determines the composition of the murine gut microbiome independently of obesity Gastroenterology 2009 137 1716 1724 10.1053/j.gastro.2009.08.042 PMC2770164 19706296

[B33] Backhed F Changes in intestinal microflora in obesity: cause or consequence? J Pediatr Gastroenterol Nutr 2009 48 Suppl 2 S56 S57 10.1097/MPG.0b013e3181a11851 19300127

[B34] Turnbaugh PJ Bäckhed F Fulton L Gordon JI Diet-induced obesity is linked to marked but reversible alterations in the mouse distal gut microbiome Cell Host Microbe 2008 3 213 223 10.1016/j.chom.2008.02.015 PMC3687783 18407065

[B35] Damaskos D Kolios G Probiotics and prebiotics in inflammatory bowel disease: microflora "on the scope" Br J Clin Pharmacol 2008 65 453 467 10.1111/j.1365-2125.2008.03096.x PMC2291386 18279467

[B36] Bäckhed F Manchester JK Semenkovich CF Gordon JI Mechanisms underlying the resistance to diet-induced obesity in germ-free mice Proc Natl Acad Sci 2007 104 979 984 10.1073/pnas.0605374104 PMC1764762 17210919

[B37] Ley RE Backhed F Turnbaugh P Lozupone CA Knight RD Gordon JI Obesity alters gut microbial ecology Proc Natl Acad Sci 2005 102 11070 11075 10.1073/pnas.0504978102 PMC1176910 16033867

[B38] Turnbaugh PJ Ley RE Mahowald MA Magrini V Mardis ER Gordon JI An obesity-associated gut microbiome with increased capacity for energy harvest Nature 2006 444 1027 1031 10.1038/nature05414 17183312

[B39] Bjursell M Admyre T Goransson M Marley AE Smith DM Oscarsson J Bohlooly-Y M Improved glucose control and reduced body fat mass in free fatty acid recepto 2-deficient mice fed a high-fat diet Am J Physiol Endocrinol Metab 2011 300 E211 220 10.1152/ajpendo.00229.2010 20959533

[B40] Ley RE Turnbaugh PJ Klein S Gordon JI Microbial ecology: human gut microbes associated with obesity Nature 2006 444 1022 1023 10.1038/4441022a 17183309

[B41] Zhang H DiBaise JK Zuccolo A Kudrna D Braidotti M Yu Y Parameswaran P Crowell MD Wing R Rittmann BE Human gut microbiota in obesity and after gastric bypass Proc Natl Acad Sci USA 2009 106 2365 2370 10.1073/pnas.0812600106 PMC2629490 19164560

[B42] Turnbaugh PJ Hamady M Yatsunenko T Cantarel BL Duncan A Ley RE Sogin ML Jones WJ Roe BA Affourtit JP A core gut microbiome in obese and lean twins Nature 2009 457 480 484 10.1038/nature07540 PMC2677729 19043404

[B43] Kalliomaki M Collado MC Salminen S Isolauri E Early differences in fecal microbiota composition in children may predict overweight Am J Clin Nutr 2008 87 534 538 10.1093/ajcn/87.3.534 18326589

[B44] Collado MC IE Laitinen K Salminen S Distinct composition of gut microbiota during pregnancy in overweight and normal-weight women Am J Clin Nutr 2008 88 894 899 10.1093/ajcn/88.4.894 18842773

[B45] Wu X MC Han L Nawaz M Gao F Zhang S Yu P Molecular characterisation of the faecal microbiota in patients with type II diabetes Curr Microbiol 2010 61 69 78 10.1007/s00284-010-9582-9 20087741

[B46] Lupp C Robertson ML Wickham ME Sekirov I Champion OL Gaynor EC Finlay BB Host-mediated inflammation disrupts the intestinal microbiota and promotes the overgrowth of Enterobacteriaceae Cell Host Microbe 2007 2 119 129 10.1016/j.chom.2007.06.010 18005726

[B47] Frost WJ AJ JB W Antibiotics and animal production Microbiology of Animals and Animal Products 1991 New York: Elsevier 181 194

[B48] Lin J Effect of antibiotic growth promoters on intestinal microbiota in food animals: a novel model for studying the relationship between gut microbiota and human obesity? Front Microbiol 2011 2 53 10.3389/fmicb.2011.00053 PMC3153029 21833309

[B49] Thuny F R Casalta JP Angelakis E Habib G Raoult D Vancomycin treatment of infective endocarditis is linked with recently acquired obesity PLOS One 2010 5 e9074 10.1371/journal.pone.0009074 PMC2818846 20161775

[B50] Membrez M Blancher F Jaquet M Bibiloni R Cani PD Burcelin RG Corthesy I Macé K Chou CJ Gut microbiota modulation with norfloxacin and ampicillin enhances glucose tolerance in mice FASEB 2008 22 2416 2426 10.1096/fj.07-102723 18326786

[B51] Koo HL HL DuPont Rifaximin: a unique gastrointestinal-selective antibiotic for enteric diseases Curr Opin Gastroenterol 2010 26 17 25 10.1097/MOG.0b013e328333dc8d PMC4737517 19881343

[B52] Pimentel M Lembo A Chey WD Zakko S Ringel Y Yu J Mareya SM Shaw AL Bortey E Forbes WP Rifaximin therapy for patients with irritable bowel syndrome without constipation NEJM 2011 364 22 32 10.1056/NEJMoa1004409 21208106

[B53] Fain J Release of inflammatory mediators by human adipose tissue is enhanced in obesity and primarily by the nonfat cells: A review Mediators Inflamm 2010 10.1155/2010/513948 PMC2874930 20508843

[B54] Ding S Chi MM Scull BP Rigby R Schwerbrock NM Magness S Jobin C Lund PK High-fat diet: bacteria interactions promote intestinal inflammation which precedes and correlates with obesity and insulin resistance in mouse PLOS ONE 2010 5 e12191 10.1371/journal.pone.0012191 PMC2922379 20808947

[B55] Erridge C Attina T Spickett CM Webb DJ A high-fat meal induces low-grade endotoxemia: evidence of a novel mechanism of postprandial inflammation Am J Clin Nutr 2007 86 1286 1292 10.1093/ajcn/86.5.1286 17991637

[B56] Ghanim H Abuaysheh S Sia CL Korzeniewski K Chaudhuri A Fernandez-Real J Dandona P Increase in plasma endotoxin concentrations and the expression of toll-like receptors and suppressor of cytokine signling-3 in mononuclear cells after a high-fat, high-carbohydrate meal Diabetes Care 2009 32 2281 2287 10.2337/dc09-0979 PMC2782991 19755625

[B57] Cani PD Amar J Iglesias MA Poggi M Knauf C Bastelica D Neyrinck AM Fava F Tuohy KM Chabo C Metabolic endotoxemia initiates obesity and insulin resistance Diabetes 2007 56 1761 1772 10.2337/db06-1491 17456850

[B58] Anderson PD Mehta NN Wolfe ML Hinkle CC Pruscino L Comiskey LL Tabita-Martinez J Sellers KF Rickels MR Ahima RS Innate immunity modulates adipokines in humans J Clin Endocrinol Metab 2007 92 2272 2279 10.1210/jc.2006-2545 17374708

[B59] Vijay-Kumar M Aitken JD Carvalho FA Cullender TC Mwangi S Srinivasan S Sitaraman SV Knight R Ley RE Gewirtz AT Metabolic syndrome and altered gut microbiota in mice lacking Toll-like receptor 5 Science 2010 328 228 231 10.1126/science.1179721 PMC4714868 20203013

[B60] Katz KD Hollander D Vadheim CM McElree C Delahunty T Dadufalza VD Krugliak P Rotter JI Intestinal permeability in patients with Crohn's disease and their healthy relatives Gastroenterology 1989 97 4 927 931 10.1016/0016-5085(89)91499-6 2506103

[B61] Gribar SC Richardson WM Sodhi CP Hackam DJ No longer an innocent bystander: epithelial toll-like receptor signaling in the development of mucosal inflammation Mol Med 2008 14 9-10 645 659 10.2119/2008-00035.Gribar PMC2435494 18584047

[B62] Bruewer M Luegering A Kucharzik T Parkos CA Madara JL Hopkins AM Nusrat A Proinflammatory cytokines disrupt epithelial barrier function by apoptosis independent mechanisms J Immunol 2003 171 6164 6172 10.4049/jimmunol.171.11.6164 14634132

[B63] Ohland CL Macnaughton WK Probiotic bacteria and intestinal epithelial barrier function Am J Physiol Gastrointest Liver Physiol 298 6 G807 819 10.1152/ajpgi.00243.2009 20299599

[B64] Bixquert Jimenez M Treatment of irritable bowel syndrome with probiotics. An etiopathogenic approach at last? Rev Esp Enferm Dig 2009 101 8 553 564 10.4321/s1130-01082009000800006 19785495

[B65] Guyonnet D Chassany O Ducrotte P Picard C Mouret M Mercier CH Matuchansky C Effect of a fermented milk containing Bifidobacterium animalis DN-173 010 on the health-related quality of life and symptoms in irritable bowel syndrome in adults in primary care: a multicentre, randomized, double-blind, controlled trial Aliment Pharmacol Ther 2007 26 475 486 10.1111/j.1365-2036.2007.03362.x 17635382

[B66] Kondo S Xiao J-Z Satoh T Odamaki T Takahashi S Sugahara H Yaeshima T Iwatsuki K Kamei A Abe K Antiobesity effects of Bifidobacterium breve strain B-3 supplementation in a mouse model with high-fat diet-induced obesity Biosci, Biotechnol, Biochem 2010 74 1656 1661 10.1271/bbb.100267 20699581

[B67] Kunze WA MY Wang B Huizinga JD Ma X Forsythe P Bienenstock J Lactobacillus reuteri enhances excitability of colonic AH neurons by inhibiting calcium-dependent potassium channel opening J Cell Mol Med 2009 13 2261 2270 10.1111/j.1582-4934.2009.00686.x PMC6529958 19210574

[B68] Ma X MY Wang B Huizinga JD Bienenstock J Kunze W Lactobacillus reuteri ingestion prevents hyperexcitability of colonic DRG neurons induced by noxious stimuli Am J Physiol Gastrointest Liver Physiol 2009 296 G868 875 10.1152/ajpgi.90511.2008 19179624

[B69] McKeman DP FP Dinan TG Cryan JF The probiotic Bifidobacterium infantis 35624 displays visceral antinociceptive effects in the rat Neurogastroenterol Motil 2010 22 1029 1035 10.1111/j.1365-2982.2010.01520.x 20518856

[B70] Bar F VKH Roblick U Bruch HP et al Cell-free supernatants of Escherichia coli Nissle 1917 modulate human colonic motility; evidence from an in vitro organ bath study Neurogastroenterol Motil 2009 21 559 566 10.1111/j.1365-2982.2008.01258.x 19220758

[B71] Wang B MY Diorio C Wang L Huizinga JD Bienenstock J Kunze W Lactobacillus reuteri ingestion and IK(Ca) channel blockade have similar effects on rat colon motility and myenteric neurons Neurogastroenterol Motil 2010 22 98 107 10.1111/j.1365-2982.2009.01384.x 19788711

[B72] Levy RL Linde JA Feld KA Crowell MD Jeffery RW The association of gastrointestinal symptoms with weight, diet, and exercise in weight-loss program participants Clin Gastroenterol Hepatol 2005 3 992 996 10.1016/s1542-3565(05)00696-8 16234045

[B73] Talley NJ Howell S Poulton R Obesity and chronic gastrointestinal tract symptoms in young adults: a birth cohort study Am J Gastroenterol 2004 99 1807 1814 10.1111/j.1572-0241.2004.30388.x 15330923

[B74] Verdich C Madsen JL Toubro S Buemann B Holst JJ Astrup A Effect of obesity and major weight reduction on gastric emptying Int J Obes Relat Metab Disord 2000 24 899 905 10.1038/sj.ijo.0801250 10918538

[B75] Ashburn DD Reed MJ Gastrointestinal system and obesity Crit Care Clin 2010 26 625 627 10.1016/j.ccc.2010.06.006 20970047

[B76] Moayyedi P The epidemiology of obesity and gastrointestinal and other diseases: an overview Dig Dis Sci 2008 53 2293 2299 10.1007/s10620-008-0410-z 18636328

[B77] Choi CW Kim GH Song CS Wang SG Lee BJ IH Kang DH Song GA Is obesity associated with gastropharngeal reflux disease? World J Gastroenterl 2008 14 265 271 10.3748/wjg.14.265 PMC2675125 18186566

[B78] Dore MP Maragkoudakis E Fraley K Pedroni A Tadeu V Realdi G Graham DY Delitala G Malaty HM Diet, lifestyle, and gender in gastro-esophageal reflux disease Dig Dis Sci 2008 53 2027 2032 10.1007/s10620-007-0108-7 18030622

[B79] Dickman R Feroze H Fass R Gastroesophageal reflux disease and irritable bowel syndrome: a common overlap syndrome Curr Gastroenterol Rep 2006 8 261 265 10.1007/s11894-006-0045-1 16886306

[B80] Nocon M Labenz J Jaspersen D Meyer-Sabellek W Stolte M Lind T Malfertheiner P Willich SN Association of body mass index with heartburn, regurgitation and esophagitis: results of the progression of gastroesophageal reflux disease study J Gastroenterol Hepatol 2007 22 1728 1731 10.1111/j.1440-1746.2006.04549.x 17914941

[B81] Schmulson M Pulido D Escobar C Farfan-Labone B Lopez-Alvarenga JC Heartburn and other related symptoms are independent of body mass index in irritable bowel syndrome Rev Exp Enferm Dig (Madrid) 2010 102 229 233 10.4321/s1130-01082010000400002 20486744

[B82] John BJ Irukulla S Abulafi AM Kumar D Mendall MA Systematic review: adipose tissue, obesity and gastrointestinal diseases Aliment Pharmacol Ther 2006 23 1511 1523 10.1111/j.1365-2036.2006.02915.x 16696799

[B83] Bassett JK Severi G English DR Bagietto L Krishnan K Hopper JL Giles GG Body size, weight change, and risk of colon cancer Cancer Epediemiol Biomarkers Prev 2010 19 2978 2986 10.1158/1055-9965.EPI-10-0543 20870733

[B84] Stachowicz M Mazurek U Nowakowska-Zajdel E Niedworok E Fatyga E Muc-Wierzgon M Leptin and its receptors in obese patients with colorectal cancer J Biol Regul Homeost Agents 2010 24 287 295 20846476

[B85] Catalán V Gómez-Ambrosi J Rodríguez A Ramírez B Silva C Rotellar F Hernández-Lizoain JL Baixauli J Valentí V Pardo F Up-regulation of the novel proinflammatory adipokines lipocalin-2, chitinase-3 like 1 and osteopontin as well as angiogenic-related factors in visceral adipose tissue of patients with colon cancer J Nutr Biochem 2010 10.1016/j.jnutbio.2010.04.015 20961744

[B86] Padidar S Farquharson AJ Williams LM Kelaiditi E N. H Arthur JR Drew JE Leptin upregulates pro-inflammatory cytokines in discrete cells within mouse colon J Cell Physiol 2010 10.1002/jcp.22546 21520064

[B87] Pendyala S Neff LM Suarez-Farinas M Holt PR Diet-induced weight loss reduces colorectal inflammation: implications for colorectal carcinogenesis Am J Clin Nutr 2010 10.3945/ajcn.110.002683 PMC3021422 21147860

[B88] Bonilla S Wang D Saps M Obesity predicts persistence of pain in children with functional gastrointestinal disorders Int J Obes (Lond) 2010 10.1038/ijo.2010.245 21079618

[B89] Venkatasubramani N Telega G Werlin SL Obesity in pediatric celiac disease J Pediatr Gastroenterol Nutr 2010 51 295 297 10.1097/MPG.0b013e3181d1365a 20479683

[B90] Furness J The enteric nervous system: normal functions and enteric neuropathies Neurogastroenterol Motil 2008 20 Suppl 1 32 38 10.1111/j.1365-2982.2008.01094.x 18402640

[B91] Mawe GM SD Sharkey KA Plasticity of enteric nerve functions in the inflamed and postinflamed gut Neurogastroenterol Motil 2009 21 481 491 10.1111/j.1365-2982.2009.01291.x PMC2717558 19368664

[B92] Lakhan SE Kirchgessner A Neuroinflammation in inflammatory bowel disease J Neuroinflammation 2010 7 37 10.1186/1742-2094-7-37 PMC2909178 20615234

[B93] Geboes K Collins S Structural abnormalities of the nervous system in Crohn's disease and ulcerative colitis Neurogastroenterol Motil 1998 10 189 202 10.1046/j.1365-2982.1998.00102.x 9659662

[B94] Bernardini N Segnani C Ippolito C De Griogio R Colucci R Faussone-Pellegrini MS Chiarugi M Campani D Castagna M Mattii L Immunohistochemical analysis of myenteric ganglia and interstitial cells of Cajal in ulcerative colitis J Cell Mol Med 2011 10.1111/j.1582-4934.2011.01298.x PMC3823295 21426484

[B95] De Giorgio R Camilleri M Human enteric neuropathies: morphology and molecular pathology Neurogastroenterol Motil 2004 16 5 515 531 10.1111/j.1365-2982.2004.00538.x 15500508

[B96] Tornblom H Lindberg G Nyberg B Veress B Full-thickness biopsy of the jejunum reveals inflammation and enteric neuropathy in irritable bowel syndrome Gastroenterology 2002 123 6 1972 1979 10.1053/gast.2002.37059 12454854

[B97] Hoffman JM MN Sharkey KA Mawe GM The relationship between inflammation-induced neuronal excitability and disrupted motor activity in the guinea pig distal colon Neurogastroenterol Motil 2011 23 673 10.1111/j.1365-2982.2011.01702.x 21426440

[B98] Dunlop SP Jenkins D Spiller RC Distinctive clinical, psychological, and histological features of postinfective irritable bowel syndrome Am J Gastroenterol 2003 98 7 1578 1583 10.1111/j.1572-0241.2003.07542.x 12873581

[B99] Isgar B Harman M Kaye MD Whorwell PJ Symptoms of irritable bowel syndrome in ulcerative colitis in remission Gut 1983 24 3 190 192 10.1136/gut.24.3.190 PMC1419934 6826101

[B100] Hyland NP Rybicka JM Ho W Pittman QJ Macnaughton WK Sharkey KA Adaptation of intestinal secretomotor function and nutrient absorption in response to diet-induced obesity Neurogastroenterol Motil 2010 22 602 e171 10.1111/j.1365-2982.2010.01504.x 20426798

[B101] Sullivan A Nord CE Evengard B Effect of supplement with lactic-acid producing bacteria on fatigue and physical activity in patients with chronic fatigue syndrome Nutr J 2009 8 4 10.1186/1475-2891-8-4 PMC2642862 19171024

[B102] Mostoslavsky R Chua KF Lombard DB Pang WW Fischer MR Gellon L LP Mostoslavsky G Franco S Murphy MM Mills KD Genomic instability and aging-like phenotype in the absence of mammalian SIRT6 Cell 2006 124 315 329 10.1016/j.cell.2005.11.044 16439206

[B103] Xiao C Kim HS Lahusen T Wang RH Xu X Gavrilova O Jou W Gius D Deng CX SIRT6 deficiency results in severe hypoglycemia by enhancing both basal and insulin-stimulated glucose uptake in mice J Biol Chem 2010 285 36776 36784 10.1074/jbc.M110.168039 PMC2978606 20847051

[B104] Kanfi Y Peshti V Gil R Naiman S Nahum L Levin E Kronfeld-Schor N Cohen HY SIRT6 protects against pathological damage caused by diet-induced obesity Aging Cell 2010 9 162 173 10.1111/j.1474-9726.2009.00544.x 20047575

[B105] Schwer B Schumacher B Lombard DB Xiao C Kurtev MV Gao J Schneider JI Chai H Bronson RT Tsai LH Neural sirtuin 6 (Sirt6) ablation attenuates somatic growth and causes obesity PNAS 2010 107 21790 21794 10.1073/pnas.1016306107 PMC3003110 21098266

[B106] Kendrick AA Choudhury M Rahman SM McCurdy CE Friederich M Van Hove JL Watson PA Birdsey N Bao J Gius D Fatty liver is associated with reduced SIRT3 activity and mitochondrial protein hyperacetylation Biochem J 2011 433 505 514 10.1042/BJ20100791 PMC3398511 21044047

[B107] Howitz KT Bitterman KJ Cohen HY Lamming DW Lavu S Wood JG Zipkin RE Chung P Kisielewski A Zhang LL Small molecule activators of sirtuins extend Saccharomyces cerevisiae lifespan Nature 2003 425 191 196 10.1038/nature01960 12939617

[B108] Wood JG Rogina B Lavu S Howitz K Helfand SL Tatar M Sinclair D Sirtuin activators mimic caloric restriction and delay ageing in metazoans Nature 2004 430 686 689 10.1038/nature02789 15254550

[B109] Kim S Jin Y Choi Y Park T Resveratrol exerts anti-obesity effects via mechanisms involving down-regulation of adipogenic and inflammatory processes in mice Biochem Pharmacol 2011 10.1016/j.bcp.2011.03.012 21439945

[B110] Qiao I Shao J Sirt1 regulates adiponectin gene expression through Foxo1-C/EBPalpha transcriptional complex J Biol Chem 2006 281 39915 39924 10.1074/jbc.M607215200 17090532

[B111] Picard F Kurtev M Chung N Topark-Ngarm A Senawong T Machado De Oliveira R Leid M McBurney MW Guarente L Sirt1 promotes fat mobilization in white adipocytes by repressing PPAR-gamma Nature 2004 429 771 776 10.1038/nature02583 PMC2820247 15175761

[B112] Pedersen SB Olholm J Paulsen SK Bennetzen MF Richelsen B Low Sirt1 expression, which is upregulated by fasting, in human adipose tissue from obese women Int J Obes (Lond) 2008 32 1250 1255 10.1038/ijo.2008.78 18560370

[B113] Peeters AV Beckers S Verrijken A Mertens I Roevens P Peeters PJ Van Hul W Van Gaal LF Association of SIRT1 gene variation with visceral obesity Hum Genet 2008 124 431 436 10.1007/s00439-008-0567-8 18820948

[B114] Zillikens MC van Meurs JBJ Rivadeneira F Amin N Hofman A Oostra Ba Sijbrands EJ Witteman JC Pols HA van Duijn CM SIRT1 genetic variation is related to BMI and risk of obesity Diabetes 2009 58 2828 2834 10.2337/db09-0536 PMC2780870 19741164

[B115] Zhu J Yong W Wu X Yu Y Lv J Liu C Mao X Zhu Y Xu K Han X Anti-inflammatory effect of resveratrol on TNF-a-induced MCP-1 expression in adipocytes Biochem Biophys Res Commun 2008 369 471 477 10.1016/j.bbrc.2008.02.034 18291098

[B116] Pfluger PT Herranz D Velasco-Miguel S Serrano M Tschop MH Sirt1 protects against high-fat diet-induced metabolic damage Proc Natl Acad Sci 2008 105 9793 9798 10.1073/pnas.0802917105 PMC2474520 18599449

[B117] Fischer-Posovszky P Kukulus V Tews D Unterkircher T Debatin KM Fulda S Wabitsch M Resveratrol regulates human adipocyte number and function in a Sirt1-dependent manner Am J Clin Nutr 2010 92 5 15 10.3945/ajcn.2009.28435 20463039

[B118] Yoshizaki T Milne JC Imamura T Schenk S Sonoda N Babendure JL Lu JC Smith JJ Jirousek MR Olefsky JM Sirt1 exerts anti-inflammatory effect and improves insulin sensitivity in adipocytes Mol Cell Biol 2009 29 1363 1374 10.1128/MCB.00705-08 PMC2643824 19103747

[B119] Ramadori G Lee CE Bookout AL Lee S Williams KW Anderson J Elmquist JK Coppari R Brain SIRT1: anatomical distribution and regulation by energy availability J Neurosci 2008 28 9989 9996 10.1523/JNEUROSCI.3257-08.2008 PMC2578850 18829956

[B120] Cakir I Perello M Lansari O Messier NJ Vaslet CA Nillni EA Hypothalmaic SIRT1 regulates food intake in a rodent model system PLOS ONE 2009 4 e8322 10.1371/journal.pone.0008322 PMC2790615 20020036

[B121] Ramadori G Fujikawa T Fukuda M Anderson J Morgan DA Mostoslavsky R Stuart RC Perello M Vianna CR Nillni EA SIRT1 deacetylase in POMC neurons is required for homeostatic defenses against diet-induced obesity Cell Metab 2010 12 10.1016/j.cmet.2010.05.010 PMC2904327 20620997

[B122] Bereswill S Muñoz M Fischer A Plicket R Haag LM Otto B Kühl AA Loddenkemper C Göbel UB Heimesaat MM Anti-inflammatory effects of resveratrol, curcumin and simvastatin in acute small intestinal inflammation PLOS One 2010 5 e15099 10.1371/journal.pone.0015099 PMC2997083 21151942

[B123] Hofseth LJ Singh UP Singh NP Nagarkatti M Nagarkatti PS Taming the beast within: resveratrol suppresses colitis and prevents colon cancer Aging 2010 2 183 184 10.18632/aging.100143 PMC2881508 20436227

[B124] Larrosa M Yanez-Gascon MJ Selma MV Gonzalez-Sarrias A Toti S Ceron JJ Tomas-Bareran F Dolara P JC E Effect of low dose of dietary resveratrol on colon microbiota, inflammation and tissue damage in a DSS-induced colitis rat model J Agric Food Chem 2009 57 2211 2220 10.1021/jf803638d 19228061

[B125] Cani PD Delzenne NM Involvement of the gut microbiota in the development of low grade inflammation associated with obesity: focus on this neglected partner Acta Gastroenterol Belg 2010 73 267 269 20690567

[B126] Vanamala J Tarver CC Murano PS Obesity-enhanced colon cancer: functional food compounds and their mechanisms of action Curr Cancer Drug Targets 2008 8 611 633 10.2174/156800908786241087 18991570

[B127] Bernard CE Gibbons SJ Gomez-Pinilla PJ Lurken MS Schmalz PF Roeder JL Linden D Cima RR Dozois EJ Larson DW Effect of age on the enteric nervous system of the human colon Neurogastroenterol Motil 2009 21 746 747 10.1111/j.1365-2982.2008.01245.x PMC2776702 19220755

[B128] Liu M Kirchgessner AL Agonist- and reflex-evoked internalization of metabotropic glutamate receptor 5 in enteric neurons J Neurosci 2000 20 3200 3205 10.1523/JNEUROSCI.20-09-03200.2000 PMC6773146 10777784

